# Multitemporal analysis of land subsidence induced by open-pit mining activity using improved combined scatterer interferometry with deep learning algorithm optimization

**DOI:** 10.1038/s41598-024-56347-0

**Published:** 2024-03-15

**Authors:** Muhammad Fulki Fadhillah, Wahyu Luqmanul Hakim, Seul-ki Lee, Kwang-Jae Lee, Seung-Jae Lee, Sung-Ho Chae, Hoonyol Lee, Chang-Wook Lee

**Affiliations:** 1 Department of Science Education, Kangwon National University, 1 Gangwondaehak-Gil, Chuncheon-Si, Gangwon-Do 24341 Republic of Korea; 2 Satellite Application Division, Korea Aerospace Research Institute, Daejeon, Republic of Korea; 3 Department of Geophysics, Kangwon National University, 1 Gangwondaehak-Gil, Chuncheon-Si, 24341 Republic of Korea

**Keywords:** Natural hazards, Solid Earth sciences

## Abstract

Mine operational safety is an important aspect of maintaining the operational continuity of a mining area. In this study, we used the InSAR time series to analyze land surface changes using the ICOPS (improved combined scatterers with optimized point scatters) method. This ICOPS method combines persistent scatterers (PS) with distributed scatterers (DS) to increase surface deformation analysis’s spatial coverage and quality. One of the improvements of this study is the use of machine learning in postprocessing, based on convolutional neural networks, to increase the reliability of results. This study used data from the Sentinel-1 SAR C-band satellite during the 2016–2022 observation period at the Musan mine, North Korea. In the InSAR surface deformation time analysis, the maximum average rate of land subsidence was approximately > 15.00 cm per year, with total surface deformation of 170 cm and 70 cm for the eastern dumping area and the western dumping area, respectively. Analyzing the mechanism of land surface changes also involved evaluating the geological conditions in the Musan mining area. Our research findings show that combining machine learning and statistical methods has great potential to enhance the understanding of mine surface deformation.

## Introduction

Recently, the surge in industrial development based on mineral resources has led to an increase in the demand for mining commodities. This increase certainly increases the concern regarding the sustainable mining industry. One of the concerns about the impact of mining activities is the potential for environmental damage, such as landslides or land subsidence^1–3^. In monitoring surface deformation caused by mining activities, various geodetic survey techniques, both land-based and remote sensing-based, have been used. Land-based methods, such as leveling and geophysical investigations, provide high-precision measurements with dense temporal sampling. However, these measurements lack spatial characterization of surface deformation due to the rarity of the measurement points^4^. The advent of satellite-based interferometric synthetic aperture radar (InSAR) techniques has brought new possibilities for monitoring surface deformation with high accuracy and unmatched spatial–temporal resolution^5^. In particular, the successful operation of the SAR Band C Sentinel-1 (S-1) satellite through the Copernicus initiative by the European Space Agency (ESA) has resulted in a wide range of InSAR applications for monitoring earthquakes, landslides, volcanic activity, urban deformation and mining activity^6,7^. Despite its success in measuring surface deformation associated with mining activity, InSAR has limitations. The land subsidence usually occurs at approximately the submeter or even meter scale in a very short period. This can lead to unwrapping phase errors or increased decorrelations^8^. In addition, mining areas are usually located in nonurban areas and even remote areas with unstable properties at all times, which impacts the availability of coherence targets that SAR observation data can identify.

Using InSAR to monitor surface deformation induced by mining activities may be a more effective tool, potentially improving sustainable mine management. Developing the pixel selection method is a key prerequisite for using the InSAR method to monitor surface deformation. Pixel selection can be divided into two types based on scattering mechanism and behaviour: persistent scatterers (PS) and distributed scatterers (DS). PS approaches focused on assessing backscattering and the phase stability of pixel targets^9–11^. The PS method’s shortcomings are the limited spatial density of pixels obtained since they are spread throughout regions of high phase stability, which is uncommon in the mining industry. Meanwhile, DS-based pixel selection increases pixel density by selecting a wide group of adjacent pixels with similar scattering mechanisms and evaluating them using statistical methods. In addition, the development of DS lies in determining DS using statistical methods to analyze spatial homogeneous pixels (SHP), such as the Kolmogorov–Smirnov, Anderson‒Darling^12^, and GLR tests^13^. Then, coherence analysis is performed on the data to obtain pixels with high coherence and determine the DS. Using PS and DS points in time series analysis allows points with consistent and widely distributed deformation change to be identified^14,15^.

One approach to improve InSAR measurement is utilizing multitemporal analysis to minimize temporal restrictions and other disruptions. Furthermore, the multitemporal InSAR approaches are the small baseline subset approach (SBAS), which limits the temporal baseline, and the Stanford method for persistent scatterers (StaMPS)^11,16,17^. By looking at trends in surface deformation over the long term, we can observe changes that occur over time, such as subsidence, lateral movement, or an increase in deformation^18,19^. This information can be invaluable for monitoring and managing mining areas, thereby assisting geotechnical risk mitigation and planning of more sustainable mining activities^20^. Through this study, our objective is to enhance the comprehension of surface deformation linked to mining operations and make a valuable contribution to advancing the application of combined scatterer interferometry with optimized point scatterers (ICOPS) time series InSAR methodology in mining regions. The primary enhancement of the ICOPS is the implementation of a machine learning algorithm to enhance the measurement points and generate a spatial clustering map^21–23^. The results of this study are expected to provide a strong basis for more effective decision-making in sustainable and environmental mining management.

In this study, we employ the ICOPS time series InSAR method to assess the trend of surface deformation in a mining region where direct measurements are challenging to acquire. Open-pit mining methods are utilized in the Musan mining region of North Korea, which is recognized as one of the largest iron deposits on the Korean Peninsula^24^. The Musan mining area, North Korea, was chosen as the study area for applying the multitemporal InSAR ICOPS method. The data used are SAR data with C-band data from the Sentinel-1 satellite with an analytical period of 2016–2022. For monitoring and analyzing surface deformation caused by mining operations, the InSAR time series application in mining regions has considerable potential. In this study, we employ the ICOPS time series InSAR method to assess the trend of surface deformation in a mining region where direct measurements are challenging to acquire. Moreover, employing this method allows us to enhance our comprehension of the surface deformation characteristics in the mining region.

## Data and methods

### Study area

**Figure 1:**
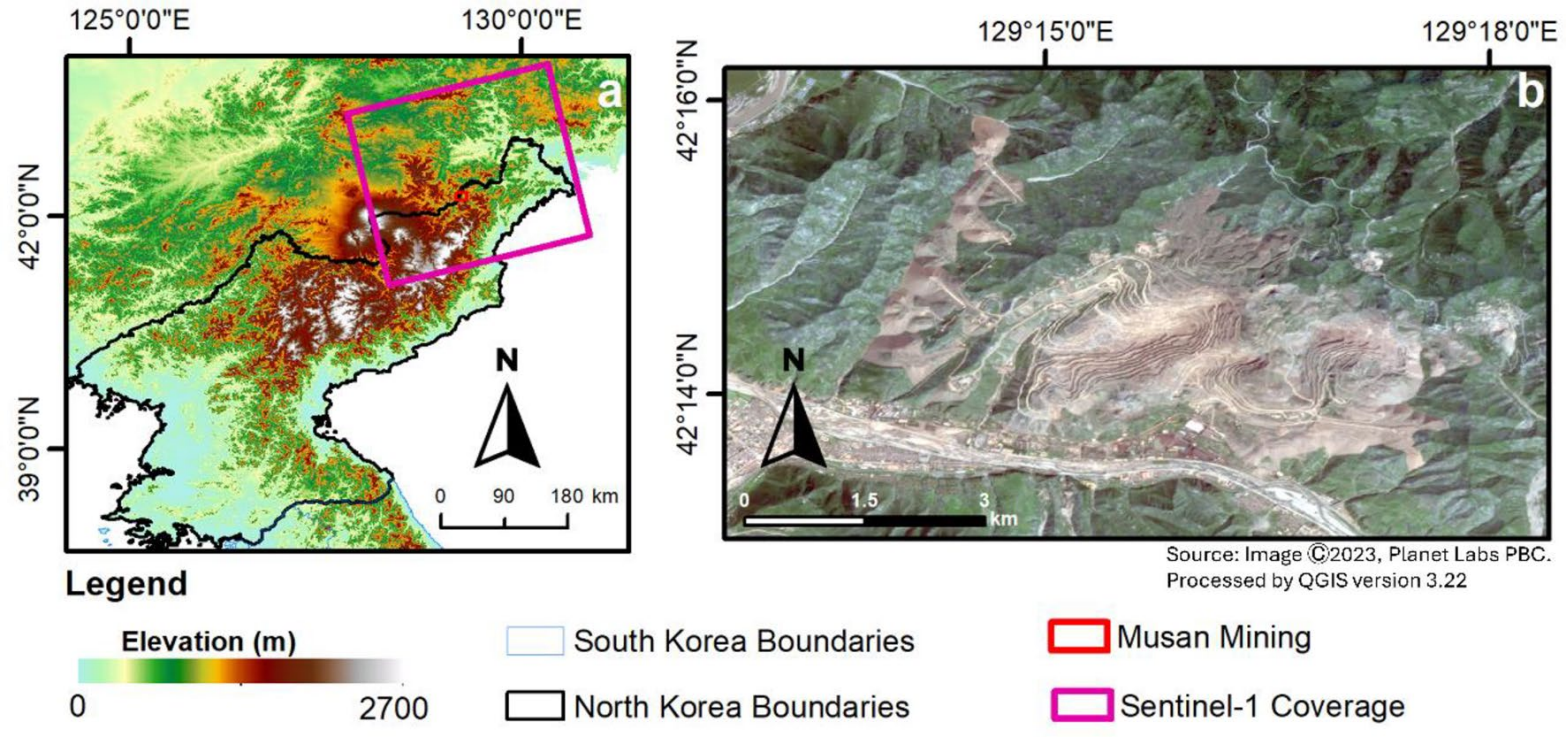
(**a**) Elevation map of North Korea, with Sentinel-1 satellite coverage and approximate location of the Musan mining area (red box) and (**b**) optical imagery of the Musan mining area from Planet Labs and processed using QGIS ver 3.22.

The Musan mine is a large-scale iron ore mining complex located in the northeast region of North Korea, near the border with China and Russia. The area’s topography is rugged mountainous terrain with elevations ranging from 400 to 1400 m above sea level. The Musan mining site in North Korea is in a mountainous region, with the mine situated in a valley surrounded by steep hills and ridges^25^. The Musan mining area is situated in the foothills of the Changbai Mountains, part of a volcanic chain that runs along the border between China and North Korea. The area is dissected by several major rivers, including the Tumen River, which flows northward into the Sea of Japan^26^. Figure 1 shows the location of Musan mining in topographical images. 1a and optical imagery around the mining site in Fig. 1b.

The Musan mine is an open-pit mine covering an area of approximately 10 square kilometers, with iron ore extracted from the surface using heavy machinery, such as excavators and trucks^27^. The terrain around the Musan mining area is heavily forested, with dense stands of coniferous trees covering much of the landscape. The soils in the area are generally thin and rocky, with shallow topsoil overlying bedrock^25^. The topography of the Musan mining site poses various challenges for mining operations, including steep slopes, rugged terrain, and difficult access to site^24^. Overall, the topographical characteristics of the Musan mining site present both challenges and opportunities for mining operations, with the rugged terrain and complex topography requiring specialized equipment and techniques to extract the iron ore while minimizing the impact on the surrounding environment^28^.

### Datasets

**Figure 2:**
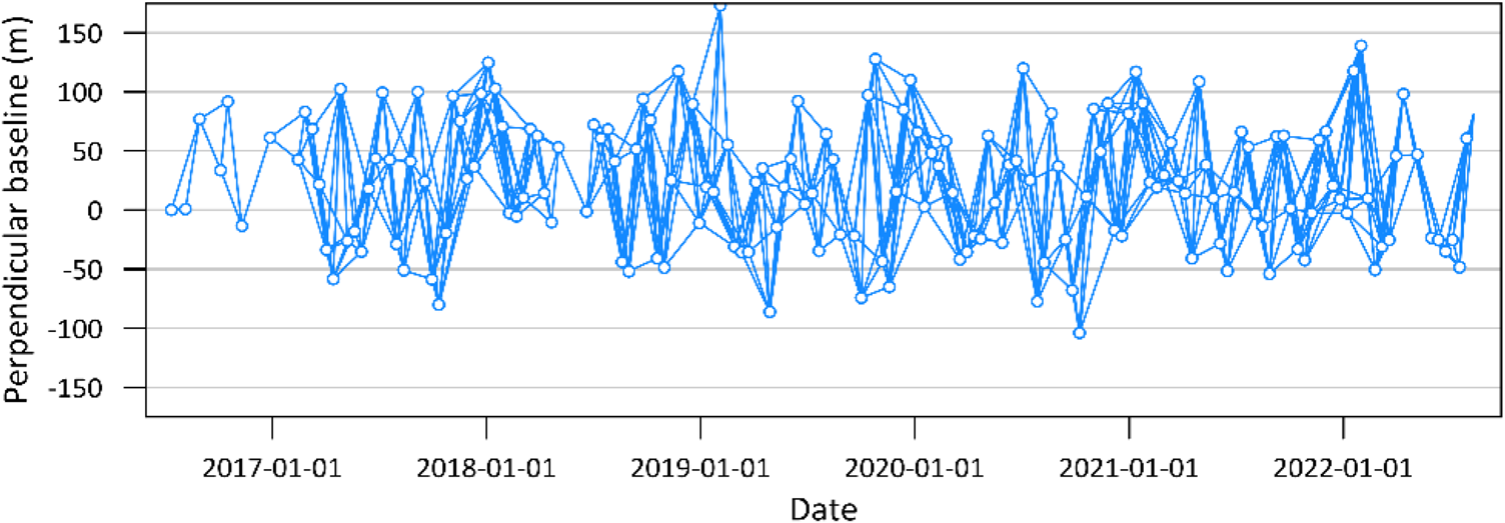
Perpendicular baseline from SAR data used in this study.

Sentinel-1 is a remote sensing radar satellite that uses a synthetic aperture radar (SAR) method to acquire information about the earth’s surface. These SAR data are used to map and monitor the Earth’s surface using VV (vertical–vertical) polarization, and the data covers the period from 13 July 2016 to 11 August 2022, which collected a number of images that were used in this study about 152 SAR images. We use the main reference from 01 September 2019 and the maximum temporal difference of 60 days on the generating interferogram, which forms 659 interferograms. For this study, we define the baseline of the repeated satellite imagery as having a maximum perpendicular baseline of approximately 200 m, as shown in Fig. 2, which is the relative distance between two different radar observations. SAR data enables accurate deformation measurements on various surfaces, including areas covered with vegetation or subject to adverse weather conditions.

Digital elevation models (DEMs) in InSAR time series deformation measurements offer an important contribution to the high-precision analysis of changes in the Earth’s surface. When radar waves reach the Earth’s surface, they interact with the topography and produce an interference phase caused by surface deformation and differences in topographic elevations. Therefore, a DEM is used to estimate and remove the topographic contribution from the SAR interferometric data, thereby increasing the accuracy in obtaining true deformation information. On the other hand, we also try to assess the change in elevation in the Musan mining area by providing the DEM for several generations in Table 1. Elevation changes are used to analyze the changes in topography at the mining site to gain more information about mining activity.

**Table 1:** The digital elevation model is used to analyze the elevation change in the Musan mining area.

	Date acquisition	Satellite	Resolution
SRTM DEM	February 2000	Shuttle Radar Topography Mission	30 m
ALOS Global Digital Surface Model	January, 2006–January 2011	ALOS-1	30 m
Copernicus DEM	January, 2011–January 2015	TanDEM-X	30 m

### ICOPS method

**Figure 3:**
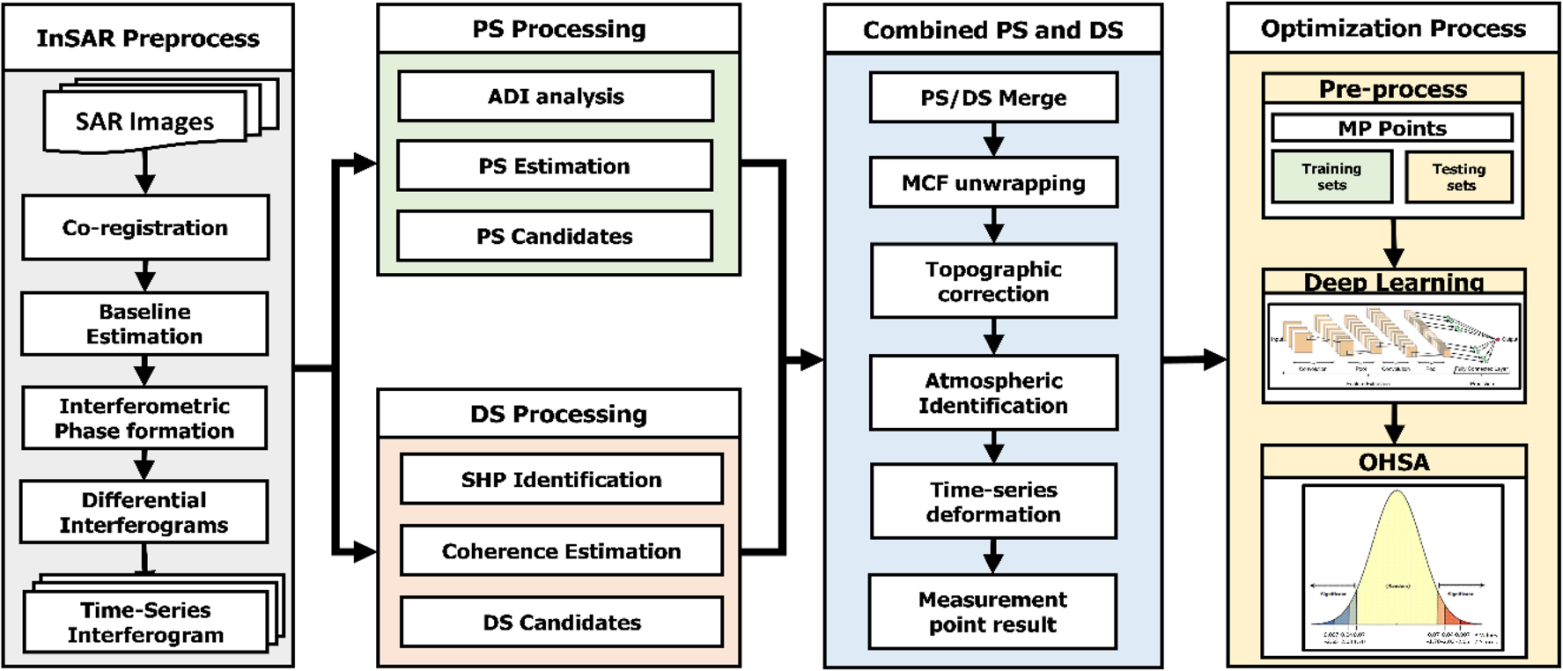
Research framework of the ICOPS combining PS and DS processing and optimization processes using convolutional neural networks.

In this study paper, the InSAR ICOPS time series analysis method was developed to monitor spatiotemporal surface deformation in the Musan mining area, North Korea, for the 2016–2022 observation period. Persistent and distributed scatterers in time series analysis provide a sufficient combination of measurement points (MPs) for spatial coverage. Therefore, developing information extraction from MPs is required to obtain reliable time series information. The time series analysis is optimized using a deep learning algorithm, the convolutional neural network (CNN), with a combination of algorithm optimization. The further framework of this study of applying the ICOPS for time series analysis is shown in Fig. 3.

### Preprocessing-InSAR

In preprocessing the SAR data, we use 168 single-look complex images cropped according to the needs of the study area. The maximal temporal baseline is 60 days to form an interferogram with a maximum perpendicular baseline of 200 m. The coregistration process is carried out to match the secondary image to the reference image in subpixel accuracy to form an interferogram^29^. To increase the level of image coherence and efforts to increase the signal-to-noise ratio and process efficiency on large-scale datasets, the multilook technique is applied to the range and azimuth directions. To obtain differential interferometric SAR (DInSAR), the topographical phase is subtracted from the interferogram with an external reference DEM database^30^; during this step, we use GAMMA software.

### PS/DS point identification

Persistent scatterers (PS) can be identified using amplitude-based measures that describe temporal or spatial consistency. PS have characteristics corresponding to phase stability and high coherence, mostly found on human-made objects^31^. The temporal stability of a resolution can be described by the amplitude dispersion (${D}_{a}$)^32^, calculated using Eq. (1).

1 $${D}_{a}=\frac{{\sigma }_{a}}{{\mu }_{a}}$$

The equation uses ${\sigma }_{a}$ to represent the standard deviation of the amplitudes of the resolution for the interferogram stack and ${\mu }_{a}$ to represent the average amplitude (${\mu }_{a}$) of the resolution for the interferogram stack.

PS identification was carried out using the StaMPS method as a basic algorithm to identify the characteristics of pixels with persistent scattering. This algorithm consists of four main steps. First, an interferogram is combined to increase the resolution in scatter identification. Next, the phase stability of each resolution is estimated using a combination of amplitude and phase measurements. Initial candidates are selected based on amplitude dispersion, and then the phase terms are corrected using a weighted iterative loop to reduce the phase noise. The PS can be selected by setting a suitable threshold for phase stability, depending on the desired false-positive level. In the final stage, the deformation estimation is obtained by unwrapping the phase and compensating for the remaining phase noise using a combination of spatial and temporal filters^33^.

The DS (distributed scatterers) point’s determination begins with applying the SHP (spatially homogeneous pixels) identification algorithm to determine the similarity between neighboring pixels and central pixels using statistical inference. At this stage, we use a fast statistical homogeneous pixel selection algorithm, FaSHPS (fast statistical homogeneous pixel selection), based on likelihood ratio test (LRT) analysis to determine SHP^34^. We set a search window of 15 × 15 pixels and a significance level 0.05. Determining whether a DS candidate will be used as a DS pixel depends on the quality of the optimal phase estimation of the candidate^35^. Therefore, the fit between the original interferometric phase and the optimized phase is used as an evaluation index to measure the quality of the optimized phase^36^. The goodness-of-fit index, called temporal coherence (${\gamma }_{TC}$), is calculated using Eq. (2).

2 $${\gamma }_{TC}=\frac{2}{N(N-1)}Re\sum_{m=1}^{N}\sum_{n=m+1}^{N}{e}^{j{\varphi }_{m,n}}{e}^{-j({\theta }_{m}-{\theta }_{n})}$$

It uses ${\varphi }_{m,n}$ to represent the original interferometric phase between the m_th_ and nt_h_ acquisitions. In contrast, theta ${\theta }_{m}$ and ${\theta }_{n}$ represent the optimized phases. Only DS candidates (over 20 SHP) with a ${\gamma }_{TC}$ value greater than 0.5 are considered high-quality DS pixels. Then, PS candidate pixels (less than 20 SHP) are filtered using an amplitude dispersion threshold of 0.4. In the final step, high-quality DS and filtered PS pixels are combined to form the combined PS/DS measurement process. This set of pixels is used to obtain the InSAR deformation time series in the next stage.

ICOPS performs a time series analysis using interference based on different images. The singular value decomposition (SVD) method is used in conventional differential interference processing for the interference pairs in the set. Each phase change is related to a certain image deformation. To eliminate atmospheric influences and the resulting deformations, temporal low-pass filtering, and spatial domain high-pass filtering are performed^37,38^. At this stage, the time series deformation is obtained from the PS/DS point and converted into a measurement point (MP).

### Optimization process

In InSAR time series applications, CNNs can study complex patterns and relationships between time series data to identify features such as deformation changes, velocities, and deformation patterns related to geological processes and environmental changes. CNNs can automatically extract important features from InSAR time series data using convolution and pooling layers^39^. The measurement points that have been identified are optimized using the CNN algorithm to obtain a better level of measurement reliability. The MP dataset is analyzed for correlation coefficients and linearity to obtain information in machine learning training. For the structure of the network, the CNN algorithm was based on the 20 convolutional layers with the rectified linear unit (ReLU) and the fully connected layers with the number of filter 32 and the filter size 7 × 7, then the activation function based on the sigmoid function on the network. For the training and test sample, we selected it from the measurement point with the linear coefficients more than 0.7 of the MP datasets will be abelled as data “1”; meanwhile, the other point will be labelled as data “0”. After that, we will divide the data “1” and “0” into training and testing samples with 70:30 comparison^22^. Then, the process is carried out using several steps in the deep learning algorithm, starting from the convolution process and the pooling layer in gathering information. Then, these data are processed so that they become a prediction model. Based on this prediction model, MP points are optimized to increase their reliability.

Then, the optimization process enters the optimization phase using optimization hot-spot analysis based on Getis-Ord statistics^40,41^. The spatial cluster process uses a model that minimizes external intervention in determining its distance, where the distance is based on Moran’s global index^41^. For MP with a large annual deformation and a high statistical value (p-value = 0.9) in the context of spatial clustering, it will create deformation clusters. Meanwhile, MP whose annual deformation value with the statistical value of clustering is below the threshold will be considered in a non-significant spatial cluster deformation. This clustering also calculates MP distribution statistics to help analyze MP distribution and characteristics. This method can also minimize deformation points that are not related in terms of distance, especially at low deformation rates.

## Results

### Mean deformation analysis

**Figure 4:**
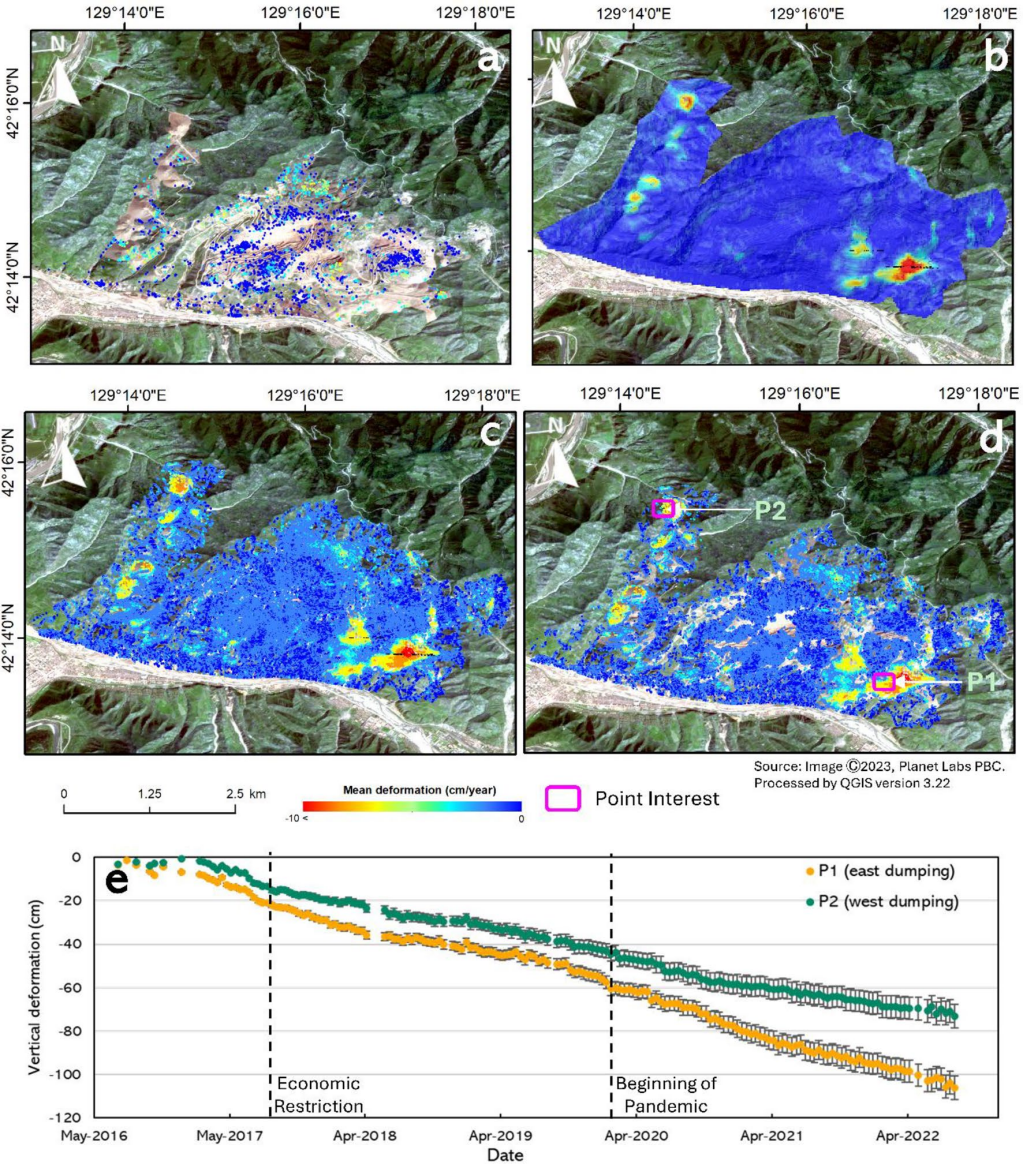
Mean deformation map retrieved from the (**a**) StaMPS processing and (**b**) SBAS processing. In addition, the result from the ICOPS method from 2016 to 2022 using Sentinel-1 datasets was present (**c**) before and (**d**) after optimization. Also, the (**e**) represents the time-series deformation graph from the P1 (eastern dump) and P2 (western dump) derived by the ICOPS method after optimization. For the map was processed using QGIS ver 3.22 and satellite imagery was derived from Planet Labs.

The ICOPS method was used to obtain deformation results in the time range from September 2016 to September 2022 based on Sentinel-1 (S-1) and Copernicus DEM (digital elevation model) data as topographic phase references. The ICOPS approach was applied to stacked interferogram datasets to analyze surface deformation at the mining site, and combining PS and DS points increased the spatial density of measurements. This section provides a mean deformation map based on persistent scatterers, specifically the StaMPS and SBAS methods, as seen in Fig. 4a,b. Surface deformation of the entire dumping area occurs within the line of sight of the satellite (LOS), and these data are converted into vertical deformation data by negligible the deformation in a horizontal direction based on the assumption the deformation occurs in a vertical direction^42^. As shown in Fig. 4, the mean deformation rate was observed in the study area, and almost all time series values were negative, indicating land subsidence. Surface deformation generally occurs in the eastern dumping areas (P1) and the western dumping areas (P2). Figure 4C shows the outcome of the ICOPS time-series processing before the optimization procedure, while Fig. 4d shows the result after the optimization process. As a result, the point discarded during the optimization process was unremarkable regarding value or spatial distribution. Then, we compared the SBAS method to ICOPS to demonstrate the optimization benefits for surface deformation at mining sites. ICOPS can reduce non-significant points that were not picked as measurement points based on the results of the optimization technique. The SBAS data reveal the mean rate of surface deformation in the mining area and the concentrations of surface deformation in the western and eastern dumping areas. Surface deformation at the dump site rises over time in response to mining process deposits. In contrast, surface changes and coherence values at the mining site influence the choice of DS point. Regional surface deformation occurred continuously at varying speeds in the dumping site, with no evidence of sudden deformation, such as collapses and landslides.

In this analysis, two main areas were identified as the areas affected by surface deformation: the eastern and western dumping areas. The eastern dumping area has experienced consistent subsidence since 2016, with a large deformation-affected area at the Musan mine complex. Based on the results, the mean deformation rate is about 15.00 cm/year in this area, with a maximum rate of deformation of 18.63 cm/year. It is known that this area is the eastern hilly area in the Musan mining complex, which was used for a long time for dumping unused extracted material. In the western dumping area, an average surface deformation of 10.20 cm/year was recorded from 2016 to 2022. The changing contours of the surface in this area almost follow the changes in the contours of the dumping area, which were made according to the conditions of the mining activity. Two points of interest in the Musan mining area were selected to gain information on surface deformation in the time series graph from 2016 to 2022, as shown in Fig. 4e.

### The trend of changes in surface deformation

**Figure 5:**
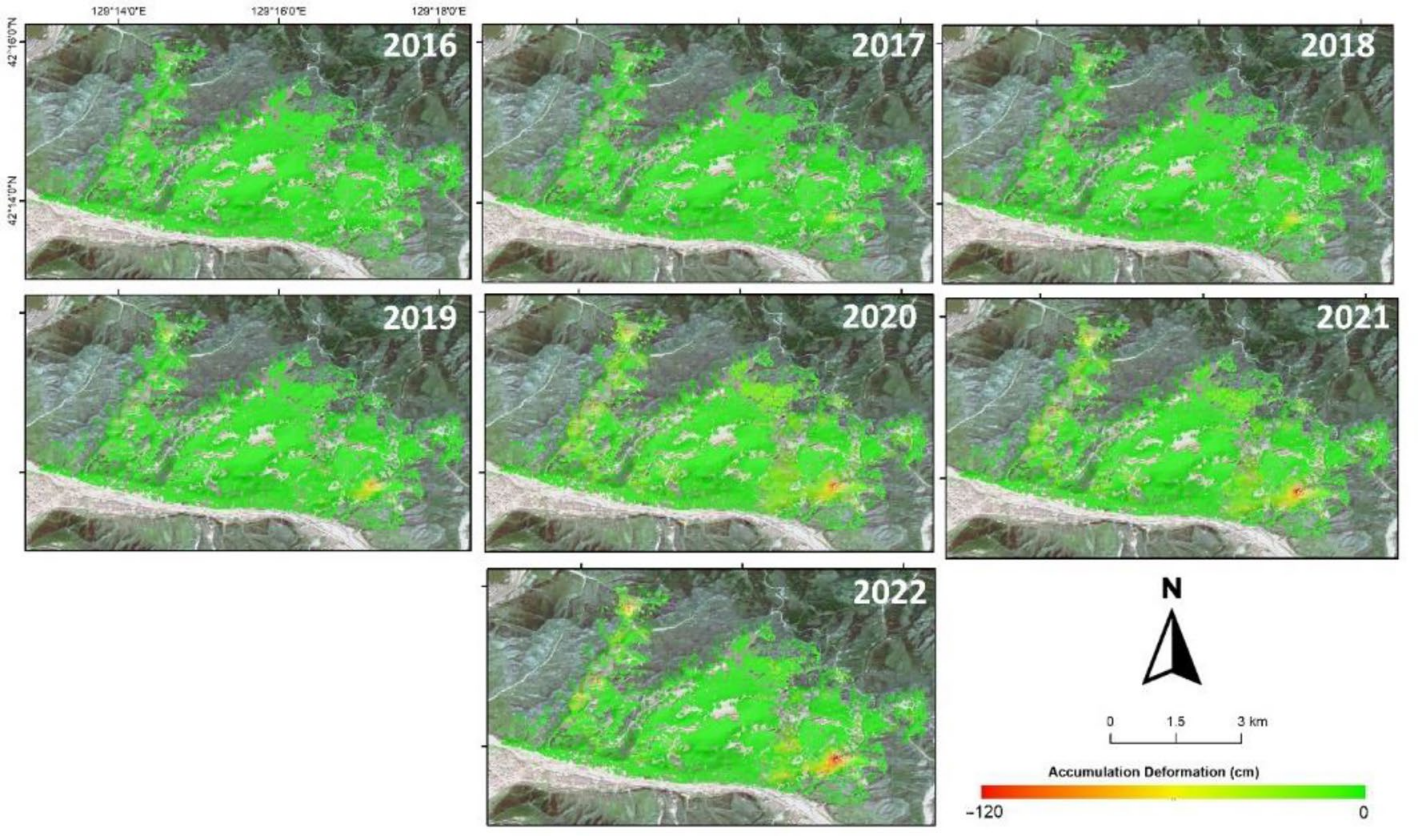
Surface deformation trend in the Musan mining area from 2016 to 2022.

This study aims to analyze the changing trend of surface deformation in the Musan mining area using InSAR time series data from 2016 to 2022. The results of the analysis show that there was a significant change in surface deformation in this area during that period. Figure 5 shows the trend of accumulated surface deformation from September 2016 to September 2022. Surface deformation in the eastern dumping area is extensive, with the highest surface deformation in all Musan mining areas, with a maximum accumulated deformation of more than 120 cm. The expansion of the eastern dumping area also contributed to the increase in surface deformation in mid-2017–2022. The eastern dumping site has operated for a longer time, which can affect the compaction of dumped material. The vertical deformations in the study area have intensified as the dump height has increased. Areas with severe deformation are mainly concentrated along the slope edges of the upper areas.

Furthermore, the western dumping area of the Musan mining complex began to show subsidence in 2018. The western dumping area experienced extensive expansion, with a large amount of stockpiled material being supplied from conveyor belts or trucks in the hilly area in approximately 2018–2019. Significant changes in surface deformation began to occur after 2019, possibly due to pressure and consolidation from heaps on the hill in this western dumping area. The change in deformation in the western dumping area can be used as a mining activity indicator. As a result, the upper part of the western dumping area, which has more surface deformation than the lower part, may indicate that the activity of dumping material was focused on the upper part rather than the lower part in this period. Moreover, most of the eroded material has been carried out of the study area for the western dumping area, and a small accumulation appears at the foot of the slope.

### Profile deformation analysis

**Figure 6:**
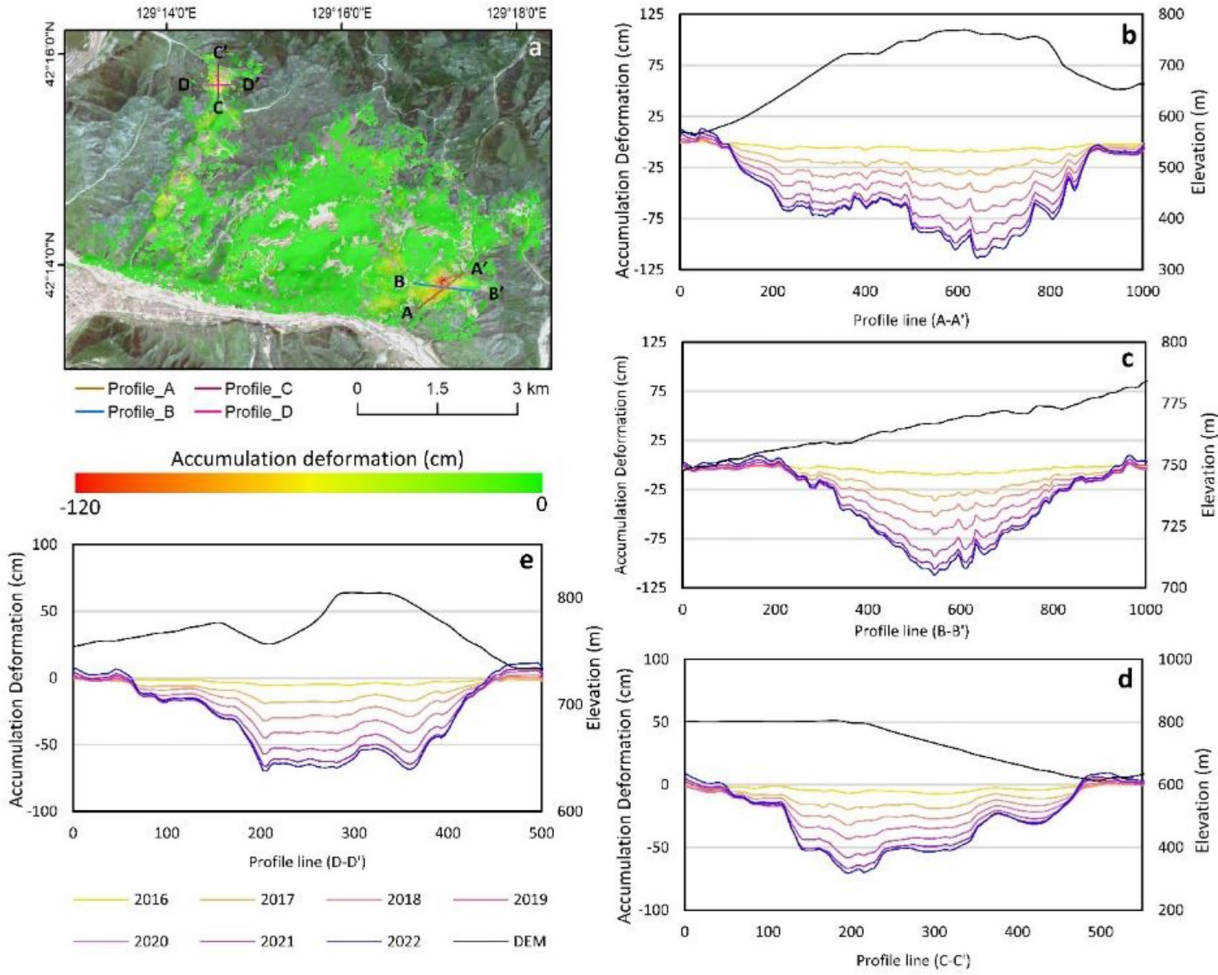
Accumulation deformation from 2016 to 2022 with profile deformation analysis of the Musan mining area (**a**) and the profile deformation results from profiles A–Aʹ (**b**) and B–Bʹ (**c**) in the eastern dumping site and profiles C–Cʹ (**d**) and D–Dʹ I in the western dumping site.

To analyze surface deformation in the Musan mining area further, deformation profiles were drawn for the eastern dumping area (area 1) and western dumping area (area 2), as shown in Fig. 6. The eastern dumping area in Figure. Profile Lines A–A represent 3b and 3cʹ and B–Bʹ, with maximum deformations reaching 115 cm and 121 cm in 2022. Moreover, the western dumping area is represented by profile Lines C–Cʹ and D–Dʹ, located in the northeastern part of the study area, and experienced maximum decreases of 63 cm and 78 cm, as shown in Fig. 3d,e. Based on the C–Cʹ and D–Dʹ profile lines, there is an indication of surface deformation along with the compaction that occurs, with uplift at the end of the deformation profile. The cumulative surface deformation curve shows a fairly consistent downward trend during the 2016–2020 phase, where the intervals of decline have a uniform magnitude in that period. However, in 2021–2022, there has been a decrease in the interval of decline, especially in 2021, due to the data not covering the full year in 2022. These results further explain surface deformation in the Musan mining area, mostly at the dumping site. The higher deformation recorded in the upper part of the western dumping site than in the lower part of this dumping site may be related to mining activity. An increase in deformation may have been induced by the expansion of the dumping process from 2018 at the western dumping site.

### Estimating changes in the volume and elevation

**Figure 7:**
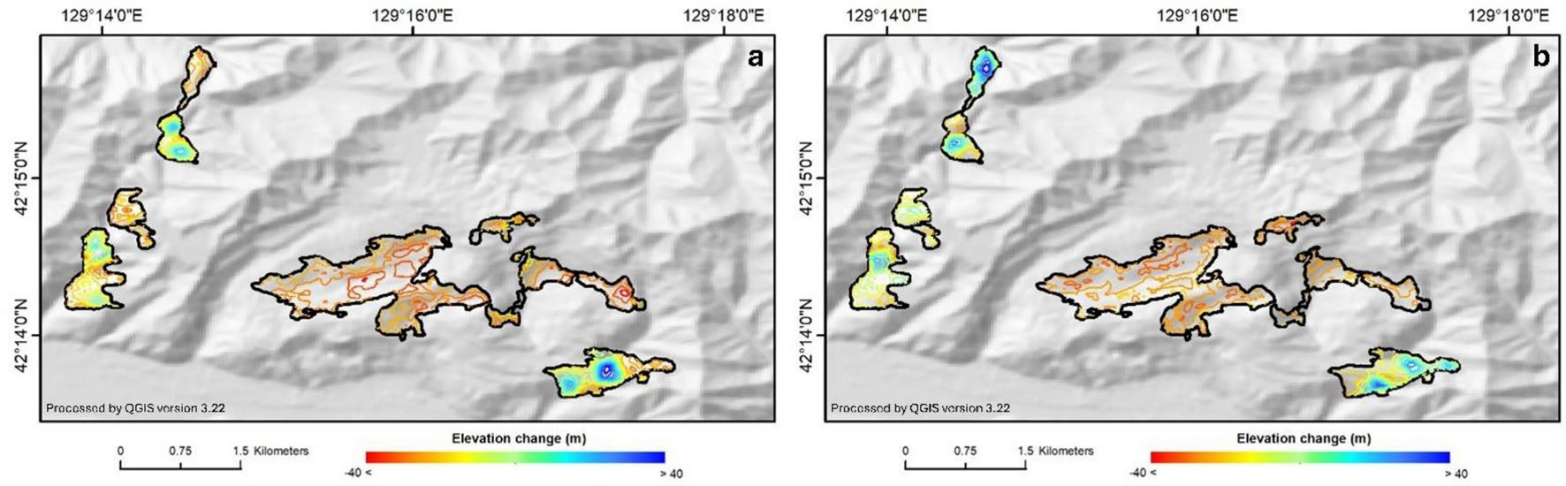
(**a**) Contour map of elevation changes from 2000 to 2010 using the SRTM DEM (2000) and ALOS World DEM (2010) and (**b**) Contour map of elevation changes from 2000 to 2010 using the ALOS DEM (2010) and Copernicus DEM (2016) was generated using QGIS version 3.22.

Besides monitoring surface deformation in recent years using InSAR, we also carried out the analysis of change elevation based on the digital elevation model (DEM). Elevation change can reflect the transformation of surface deformation in different years in the mining site. This analysis will provide valuable information that may be combined with InSAR analysis to improve understanding of the mining situation. An analysis of changes in elevation in the Musan mining area for the period of 2000–2011 using the shuttle radar topography mission (SRTM) and advanced land observing satellite (ALOS) digital elevation model (DEM) provides interesting information. Elevation changes are made by subtracting the values in the ALOS DEM from those in the SRTM DEM to determine changes, as shown on the contour map in Fig. 7a. The distribution of changes in the mining area is located in the main excavation area, which has lost elevation, with a decrease in elevation of − 40 m. There are elevation changes that are increasing in the east and west areas, known as the western dumping areas and the eastern dumping areas, respectively. The increase in elevation in this period is approximately 80 m for the western dumping area and 110 m for the eastern dumping area. Many elevation changes have occurred in the southern part of the western dumping area. Volume changes have also been used to determine changes in conditions during the 2000–2010 period, in which the mining area was 35,225,970 m^3^, while in the dumping area, values of 29,423,277 m^3^ in the western area and 17,587,725 m^3^ in the eastern area were recorded.

Between 2010 and 2016, we studied elevation changes in the Musan mining area using the Copernicus DEM and ALOS DEM. Our research found that the excavated mining area experienced a maximum height change of − 35 m, with a pattern that followed the contours of the prior elevation decrease. Additionally, the eastern dumping area experienced a height increase of 75 m, while the western dumping region experienced a height increase of 85 m. According to Fig. 7b, the contour map of elevation variations from 2010 to 2016 reveals changes in mining activities, particularly in the mining area. Although the two primary dumping places remain the most active, they are distributed in diverse directions. The eastern area has elevation changes in the northern and southern areas, whereas the western region has alterations in the north half of the dumping area. Estimation of volume changes was also carried out in the three areas of interest during the 2010–2016 observation period. The change in volume in the excavation area was − 27,425,211 m^3^, while in the western dumping area, there was a change in volume of 14,983,446 m^3^, and in the eastern dumping area, there was a change of 23,382,894.11 m^3^.

The previously reported volume changes are still raw and need adjustments to predict the iron ore mining capacity at the Musan mine. In the case of the Musan mine, only very limited geological information was provided, so information regarding the location and appearance of drilling data and development of the mine was not significant. Because the iron ore distribution pattern in the Anshan Formation is similar to that of the Musan Group at the Musan mine, the Musan Group is considered the eastern extension of the Anshan Group^25^. The most recent researcher measured the density of iron ore in the mineralized belt used at Donganshan and Qidashan in the Anshan Formation^43^. In this study, the average density of ore produced at the Musan mine was estimated to be approximately 3.4 × 10^3^ kg/m^3^ over the average density of ore produced from the Anshan Formation^43^.

The results of elevation changes based on DEM data are processed again to estimate the ore obtained from this mining activity. In the estimation process, several assumptions are applied to this calculation according to the results of the literature review that has been carried out. By applying an approximate assumption in the form of material that can be processed in the form of 0.3 volume of the total extraction, the volume difference can be converted into a predictable ore estimate that can be extracted. Then, the defined iron ore density values can be used to estimate the mass that can be extracted during that period. Based on the above assumptions, in the 2000–2011 period, there was a volume change in the mining area of 35,225,970 m^3^, and it can be estimated that the mass extracted during that period was 119.77 million tons. In the 2011–2016 period, using a difference in the volume of the mining area of 27,425,211 m^3^, it was estimated that the extracted mass was approximately 93.25 million tons. Also, we calculate the mass change on the dumping site in the 2000–2016 period, as shown in Table 2. Therefore, the estimation affected by the resolution size of the DEM led to uncertain error calculations. This calculation is an approximate calculation that requires correction in terms of rock conditions and density aspects that are more precise according to the characteristics of the Musan mine.

**Table 2:** Estimation of mass change in iron mining from change of volume derived from DEM.

	2000–2011 (SRTM–ALOS DEM)		2011–2016 (ALOS DEM–Copernicus DEM)	
	Volume change (m^3^)	Mass change (Mt)	Volume change (m^3^)	Mass change (Mt)
West Dumping	29,493,732	55.74	23,382,894	44.19
East Dumping	17,587,725	33.24	14,585,446	25.68
Excavation Site	− 35,225,970	− 119.77	− 27,425,211	− 93.25
Waste ratio		0.748		0.722

## Discussion

**Figure 8:**
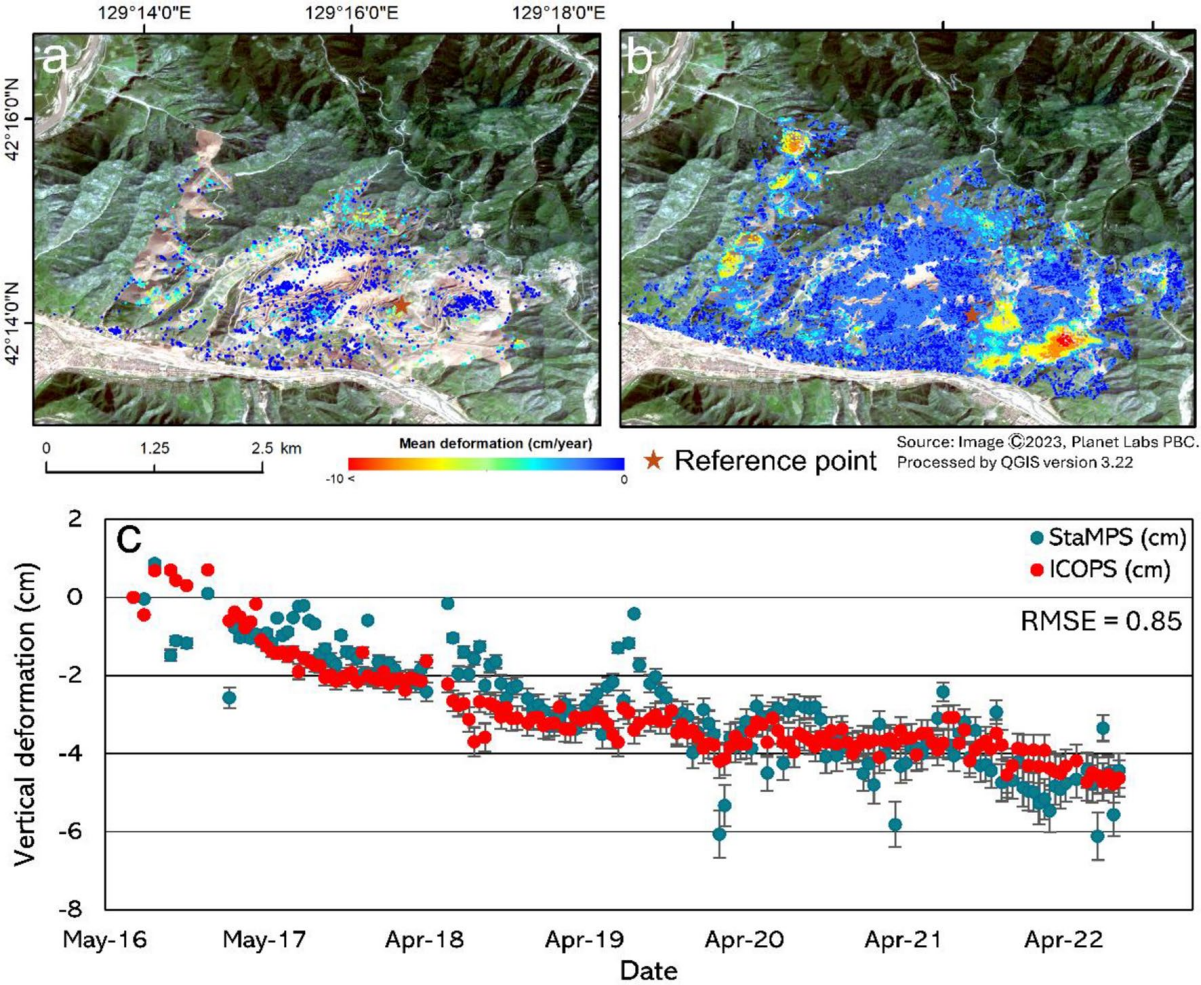
Mean deformation map derived from (**a**) StaMPS and (**b**) ICOPS overlayed satellite imagery from Planet Labs and processed using QGIS ver. 3.22. (**c**) Then, the time-series graph from the reference point is marked by a star from the StaMPS and ICOPS methods.

We applied performance analysis to evaluate subsidence measurements in the Musan mining area using ICOPS technology for multitemporal InSAR. The test is carried out by analyzing the value of the mean deformation at the measurement point produced by the two multitemporal InSAR methods, namely StaMPS and ICOPS. We investigated the time series deformation graphic measurements at points that overlap with the StaMPS method to confirm the reliability of measurements using the ICOPS method, as shown in Fig. 8. Additionally, for the StaMPS and ICOPS methods, the time series deformation measurement graphs with an estimated RMSE value of 0.85 are shown in Fig. 8c. As demonstrated by these observations, the ICOPS method measurement results are reliable when compared with other methods. Besides, the measurement points from the StaMPS and ICOPS that have coincidence or are within an area of 20 m are evaluated by cross-correlation to obtain a correlation coefficient value. Based on the results of the correlation coefficient analysis, the coefficient value of R^2^ = 0.87 m is obtained, indicating that the ICOPS method is reliable in this calculation. This performance study demonstrates that the ICOPS analysis approach is suitable for integrating other multitemporal InSAR methodologies. A comparison between the ICOPS method and geodetic data is necessary for additional analysis to assure its reliability; however, such comparisons are not feasible at this location. In addition to performance analysis, from the coverage side, ICOPS can maximize DS and PS in forming MP’s while maintaining useful surface deformation information.

This study aims to analyze the relationship between lithologic conditions and surface deformation in the Musan area and the North Korean mining area. This study area is characterized by significant lithological complexity and mining activity^44^. In general, the lithology in this area consists of igneous, metamorphic, and sedimentary deposits^24^. The dominant granitoid rocks in the area have high hardness and structural strength, so they tend to be more stable and have more controlled surface deformations. Moreover, metamorphic rocks, such as schist and gneiss, have different properties^43^. These rocks have a more complex texture and structure with diverse mineral contents. When subjected to stress and environmental changes, these properties make it susceptible to significant surface deformation. In addition, sedimentary rocks, such as sandstone, clay, and gravel, were also found in the Musan mining area. These sedimentary rocks tend to have a lower density and weaker structural strength than igneous and metamorphic rocks^45^. As a result, surface deformation in this area tends to be more significant, especially during intense mining activities.

The influence of climatic factors can also be an important consideration in the relationship between lithology and surface deformation in the Musan mining area^27^. The correlation between local climatic conditions, such as high rainfall, significant temperature changes, and surface deformation, needs to be considered. Soil erosion due to rain and thermal changes can affect slope stability and cause more significant surface deformations, especially in weaker sedimentary deposits^46^. This analysis provides a deeper understanding of the factors affecting surface deformation in the Musan mining area in North Korea. The results of this study can be used as an important foundation for sustainable mining management and future mitigation of surface deformation risks in the mining area.

## Conclusion

This study analyzes surface deformation in the Musan mining area in North Korea using the proposed InSAR time series method known as ICOPS with Sentinel-1 satellite SAR data from 2016 to 2022. Combining DS and PS boosts spatial coverage by approximately 80% compared to using PS alone, despite this investigation's lengthy temporal observation period. Deep learning algorithms are used in optimization applications to minimize interference caused by disturbances in InSAR deformation time series analysis. The examination of the time series deformation process in the Musan mining area revealed varying degrees of deformation between 2016 and 2022. Using the ICOPS approach, the excavation area experiences an average deformation magnitude of 18 cm/year, with a maximum detected deformation of 170 cm. Surface deformation in the western dumping region has accumulated to 70 cm with an average rate of 10.20 cm per year. The deformation in the dumping area is likely due to surface pressure causing soil compaction at the top, with avalanches accumulating in the valley area.

A correlation test was conducted using cross-validation between two InSAR approaches at coinciding measurement locations. The test revealed a reliable consistency between the two methods, with a correlation coefficient of R^2^ = 0.87. The study demonstrates that the ICOPS approach can enhance spatial coverage and density while maintaining measurement accuracy and reliability. Analysis of the causes of deformation is carried out by comparing the conditions of the geological area in the mining area. However, the limited sources of information about this mining area limit the analysis of the deformation. Even so, this study shows that applying the ICOPS time series method to mining areas and long temporal scales can provide reliable results. The development of deformation studies in the mining area can be carried out with comparisons and studies of the causes of deformation with better information in the future.

## Data Availability

The SAR data used for generating time-series surface deformation in the study are available at Copernicus Sentinel and processed by ESA via Alaska Satellite Facilities (https://asf.alaska.edu/).
